# 

**Published:** 1905-09-23

**Authors:** 


### NOTICE TO CORRESPONDENTS.

All MSS., Letters, Books for Review, and other matters intended for the Editor should be addressed The Editor, "The Hospital," The Hospital Buildings, 28 & 29 Southampton Street, Strand, W.C.

The Editor cannot undertake to return rejected MS., even when accompanied by stamped directed envelope.

**Notice to Advertisers and Subscribers.**

All Advertisements, Orders for Copies of The Hospital, and Business Communications should be addressed to **THE MANAGER (Not to the Editor),** The Hospital, 28 & 29 Southampton Street, Strand, London, W.C.

The Cost of Subscription, post free, is at follows:—Per week, 3½d.; per quarter, 3s. 3d.; per half-year, 6s. 6d.; per annum, 13s. Foreign and Colonial:—Per annum, 19s. 6d., payable, in all cases, in advance.

**NOTICE.—Vol. XXXVII. of "The Hospital" October 1904 to March 1905), in handsome cloth binding, will shortly be on sale at the office of this Journal, price 10s. 6d. Cases for binding the Numbers contained in this Volume 2s. Index to Vol. XXXVII., 3½d., post free.**

**The Monthly Part for September is now ready. Price 1s., by post, 1s. 3d.**

### BOOKS RECEIVED.

J. B. Lippincott Co.

"A Nurses' Handbook of Obstetrics." By J. B. Cooke, M.D.

T. Nelson and Sons.

"The Mill on the Floss."

From the Author.

"What to Do and How to Do It. A Pocket Guide to the Treatment of Natural Labour." By E. S. Payne.

Sidney Appleton.

"The Doctor Says." A book of advice for the household.

The Caxton Press, Limited.

"The Bodie Book." By Dr. W. Bodie.

John Bale, Sons, and Danielsson, Ltd.

"Pharmacopœia and Formulary of the Royal Dental Hospital of London."

A. C. Fifield.

"The Meat Fetish." By E. Crosby and E. Reclus.

H. J. Glaisher.

"A Plea for the More Energetic Treatment of the Insane." By C. Williams, L.R.C.P.

### Medical Appointments.

**William Appleyard**, M.B., B.S. Lond., M.R.C.S. Eng., L.R.C.P.Lond., House Surgeon at Bradford Infirmary. **Frederic Barker**, M.R.C.S.Eng., L.R.C.P.Lond., Resident Medical Officer at the Hospital for Epilepsy and Paralysis, Maida Vale, London, W. **Herbert Alfred De Pinna**, M.R.C.S.Eng., L.R.C.P.Lond., L.D.S.Eng., D.D.S.Ontario, Medical Officer (Ship's Surgeon) to the Peninsular and Oriental Steam Navigation Company. **Alexander Duncan**, M.B., C.M.Glasg., D.P.H.Cantab., Medical Officer of Health of Merthyr Tydvil. **Albert Irwin Eades**, L.R.C.P., L.R.C.S.Irel., Medical Superintendent of the North Riding Asylum, at Clifton, Yorkshire. **Alfred B. Gittins**, M.R.C.S.Eng., L.R.C.P.Lond., Temporary Medical Officer of part of the Whitmore District of the Newcastle Union, Staffordshire. **Janet A. S. Mouat**, M.B., Ch.B.Edin., Resident Medical Officer at the Potteries Joint Hospital, Bucknall, Staffordshire. **John O'Halloran**, L.R.C.P., L.R.C.S.Irel., Dispensary Medical Officer at Scariff, county Clare. **Charles H. Wild**, L.R.C.P.Irel., L.R.C.P.Lond., M.R.C.S.Eng., Medical Officer and Public Vaccinator for the Prescot District. **Richard T. Williams**, L.R.C.P., L.R.C.S.Edin., L.F.P.S.Glasg., Medical Officer of the Cwmavon District of the Neath Union. **Ralph Young** M.D.Durh., Medical Officer of Health of Royton, Oldham.

G H R — **Best**

We have run up this signal to our masthead, and are determined to publish

### only The Best in Nursing Literature.

### SOME NEW BOOKS ready shortly.

**SURGICAL INSTRUMENTS AND APPLIANCES USED IN OPERATIONS.**

A Classified and Illustrated List, with Explanatory Notes.

By HAROLD BURROWS, F.R.C.S.

Limp cloth, **1/6** net, **1/8** post free.

This work will prove of the utmost service as a work of reference to all Nurses.

---

**NURSING HINTS TO PROBATIONERS ON PRACTICAL WORK.**

By ANNESLEY VOYSEY.

Cloth, Illustrated, **2/-** net, **2/4** post free.

This work fills a distinct hiatus in nursing literature. It will prove of great assistance to the Nurse in the early stages of her career.

---

**MEDICAL ELECTRICITY AND THE LIGHT TREATMENT.**

By KATE NEALE,

Sister in Charge Light Department, Guy's Hospital.

Cloth, profusely Illustrated, **2/6** net, **2/9** post free.

A practical book on a subject that hitherto has been untouched from a nursing standpoint.

---

**THE NURSING OF SICK CHILDREN.**

By Dr. BURNETT.

Cloth, **1/-** net, **1/1** post free.

A valuable little treatise on the nursing of children, and should prove useful to mothers and Nurses.

### A Selection of Nursing Handbooks

**A COMPLETE HANDBOOK OF MIDWIFERY.**

By J. K. WATSON, M.D. **6/-** net, **6/4** post free.

Illustrated with 143 Diagrams. Cloth gilt.

---

**PRACTICAL GUIDE TO SURGICAL BANDAGING AND DRESSINGS.**

By WM. JOHNSON SMITH, F.R.C.S. **2/-** post free.

Suitable size for the apron pocket.

Profusely Illustrated.

---

**SURGICAL WARD-WORK AND NURSING.**

By Dr. ALEXANDER MILES, M.D. Edin.

**3/6** net, **3/10** post free.

Second Edition. Illustrated. Cloth gilt.

---

**THE EXAMINATION OF THE URINE.**

By J. K. WATSON, M.D. **1/-** net, **1/1** post free.

Cloth boards. Suitable size for the apron pocket.

---

**NURSING: ITS THEORY AND PRACTICE.**

By Dr. PERCY LEWIS, M.D. **3/6** post free.

Illustrated. Cloth gilt.

---

**ELEMENTARY ANATOMY AND SURGERY FOR NURSES.**

By WM. McADAM ECCLES, M.S., M.B. **2/6** post free.

Profusely Illustrated. Cloth.

---

**ELEMENTARY PHYSIOLOGY FOR NURSES.**

By C. F. MARSHALL, M.D., B.Sc., F.R.C.S.

**2/-** post free.

Profusely Illustrated. Cloth.

---

**GYNÆCOLOGICAL NURSING.**

By G. A. HAWKINS-AMBLER, F.R.C.S.

Cloth, **1/-** post free.

---

**The Scientific Press, Ltd.**

*Complete Catalogue sent post free on application.*

**Publishers of Nursing Text-Books, Charts, and Case-Books of all descriptions.**

**28 & 29 SOUTHAMPTON STREET, STRAND, LONDON, W.C.**

### BOOKS FOR NURSES

***Wiggins'***
**MIDWIFERY FOR MIDWIVES.** Price 3/6 net.
"Learners will find it less difficult to understand and remember than any other book on the same subject."—*British Medical Journal.*

***Calder's***
**QUESTIONS AND ANSWERS ON MIDWIFERY FOR MIDWIVES.** Price 1/6 net.
"Just what is required to help those who are not used to examinations."—*Nursing Notes.*

***Giles'***
**MENSTRUATION and its DISORDERS.** Price 2/6 net.
"There is a great deal in this book that is very interesting." *Hospital.*

***Palmer's***
**LESSONS ON MASSAGE.** Price 7/6 net.
"A most admirable and clearly expressed theory of massage." *Nursing Notes.*

***BAILLIÈRE'S MOVABLE ATLAS-MODELS AND MANIKINS.***
**THE MALE.** Price 3/6 net. **THE FEMALE.** Price 4/- net.
**THE FEMALE ORGANS OF GENERATION AND PREGNANCY.** Price 3/- net.
These Models are complete reproductions of the Human Body composed of a number of Plates which open out and fold over. They are coloured to Nature, with every bone, muscle, vein, &c. numbered, and explained in the text.

***Ellison's***
**MASSAGE FOR STUDENTS.** Price 3/6 net.
"A carefully written and very valuable book."—*Nursing Notes.*

***Mummery's***
**AFTER-TREATMENT OF OPERATIONS.** Price 5/- net.
"Should prove of great value to those who look after patients."—*Lancet.*

***Aids to***
**OBSTETRICS AND GYNÆCOLOGY.** Price 2/6 each.
"Well up-to-date and will form a valuable addition to the library." *Nursing Notes.*

***Goldie's***
**NOTES ON HOME NURSING.** Price 1/6 net.
"Contains many useful hints, including first aid in confinements."—*League News.*

**London: BAILLIÈRE, TINDALL & COX, 8 Henrietta Street, Covent Garden.**

